# Caught in the web: Exploring spider predation on bats in Europe

**DOI:** 10.1002/ece3.11474

**Published:** 2024-05-30

**Authors:** Héctor Ruiz‐Villar, Cecilia Montauban, Ana Pino‐Blanco, Elena Tena

**Affiliations:** ^1^ Independent Researcher Villablino Spain; ^2^ Department of Life Sciences Imperial College London Ascot UK; ^3^ Essex Bat Group Essex UK; ^4^ Department of Ecology and Evolution Doñana Biological Station (CSIC) Seville Spain

**Keywords:** Arachnida, Chiroptera, *Pipistrellus*, predator–prey interactions, *Steatoda nobilis*, *Eratigena*

## Abstract

The intricate interplay between predators and prey has long fascinated ecologists, with bats and their diverse prey offering insight into co‐evolutionary dynamics. While bats have evolved sophisticated strategies for prey capture, they also face predation pressure. Among their predators, spiders stand out for their diversity of predatory tactics, ranging from hunting assaults and web ensnarement to the deployment of venom. Yet, bat predation records by spiders are mostly from tropical regions, and cases remain notably scarce in temperate regions. Here, we report four new incidences of bat predation and mortality by spiders and their webs in Europe. Our observations include detailed photograph and video documentation of the first record of a spider capturing and consuming a bat pup in Spain, as well as accounts of bats entangled in spider webs on a building and inside bat boxes in the United Kingdom. These findings shed light on understudied predator–prey dynamics, offering valuable insights into spider predation on bats in European ecosystems. Our study emphasises the importance of continued research to improve our understanding of ecological interactions between these elusive and primarily nocturnal taxa.

## Graphical abstract

Despite their diverse predatory tactics, spider predation on bats remains understudied, particularly in temperate regions. This article provides detailed observations of bat predation and mortality by spiders in Europe, including the first record of a spider capturing and consuming a bat pup in Spain, as well as accounts of bats entangled in spider webs on a building and inside bat boxes in the United Kingdom. These findings offer new insights into unknown predator–prey interactions and emphasise the need for further research on the ecological relationship between these elusive nocturnal taxa.

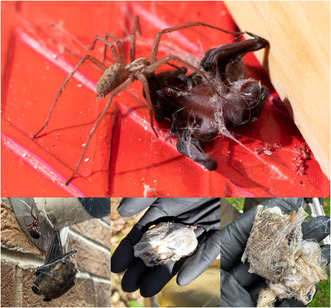

## 1 INTRODUCTION

The evolutionary arms race between bats and their prey has long captivated researchers, revealing fascinating co‐evolutionary adaptations that shape ecological interactions (Barber et al., 2022; Conner & Corcoran, 2012; Ter Hofstede & Ratcliffe, 2016). On one hand, bats exhibit diverse strategies to exploit various prey types, like frog‐eating bats able to recognise non‐poisonous anuran prey from their acoustic signals and exploit water ripples for hunting (Halfwerk et al., 2014; Tuttle & Ryan, 1981). On the contrary, prey species have evolved countermeasures, with hawkmoths, for instance, able to generate ultrasonic pulses to jam the echolocation hunting ability of bats (Barber & Kawahara, 2013; Watson, 2013). Bats are typically regarded as agile predators with few natural enemies, but accumulating evidence continues to unveil that they are also prey to many animals (Mikula et al., 2024).

A lot remains unknown about the trophic ecology and interactions of these elusive nocturnal mammals, from predator–prey links, to predator effects and tactics. Between bats and arachnids, a few bat species are renowned for eating scorpions and being immune to their venom (Holderied et al., 2011; Hopp et al., 2017). Many other species are known to feed on spiders, and the application of genetic and imaging techniques have revealed that many bat species are spider specialists (Goiti et al., 2011; Maucieri & Barclay, 2021; Schulz, 2000; Swift & Racey, 2002). Although the reverse, spiders preying on bats, might appear less common, it is conceivable given the substantial diversity and overlapping widespread distribution of both taxa, coupled with their shared nocturnal behaviour and frequent cohabitation.

Spiders prey upon a wide variety of vertebrate species, encompassing birds, frogs, lizards, fish and mammals—including bats (Nyffeler & Gibbons, 2022; Nyffeler & Vetter, 2018; Valdez, 2020), with predation defined as the intentional capture, killing and consumption of prey (Nyffeler & Knörnschild, 2013). At least 39 of the existing 135 spider families are capable of preying upon small vertebrates, with a wide variety of hunting techniques, ranging from the construction of large sticky webs to capture flying vertebrates, to active hunting and capturing of ground‐dwelling vertebrate prey (Nentwig, 1987; Nyffeler & Gibbons, 2022; Toft, 2013; World Spider Catalog, 2024). A review of bat predation by spiders shows it is predominantly reported in the Neotropics, Asia and Oceania (Nyffeler & Knörnschild, 2013). Bat‐catching spiders belong to families including Theraphosidae, Nephilidae, Araneidae, Sparassidae and sporadically, Pisauridae. Among reported cases, web‐building spiders accounted for 88% of bat predation events, while hunting spiders constituted 12% (Nyffeler & Knörnschild, 2013). Most captured bats were small aerial insectivorous vespertilionid and emballonurid species (Nyffeler & Knörnschild, 2013). Although some bats may have died from accidental entangling in spider webs, numerous reports of active predation have been observed, suggesting spider predation on bats is more widespread than previously thought (Nyffeler & Knörnschild, 2013).

However, in temperate regions, reports of spider predation on bats are notably scarce. Nyffeler and Knörnschild (2013) included only four cases, two in North America and two in Europe. The two records in Europe are from Germany and the United Kingdom, and both involve pipistrelle bats caught in webs of unidentified spiders. A subsequent case was reported in the United Kingdom by Dunbar et al. (2022), although the predation in this case has been questioned (Dool & Uhl, 2022). These occurrences all involve small *Myotis* and *Pipistrellus* species entangled in spider webs and have been interpreted as opportunistic rather than deliberate acts of predation, and possibly even cases of non‐predation death with no involvement of the spider (Dool & Uhl, 2022; Nyffeler & Knörnschild, 2013). Nonetheless, the limited documentation and understanding of these mortality events, highlights an unknown area of ecological interactions within European ecosystems.

This article aims to address this knowledge gap by reporting four new incidences of predatory interactions between bats and spiders in Europe. These cases, which include the first detailed documentation of capture, killing and consumption of a bat pup in Spain, and the first documented events of adult bats captured in spider webs inside bat boxes in the United Kingdom, shed light on this relatively obscure and understudied aspect of predator–prey dynamics. Through these findings, we seek to stimulate further research and deepen our understanding of the complexities of spider predation on bats in Europe.

## 2 METHODS AND RESULTS

**FIGURE 1:**
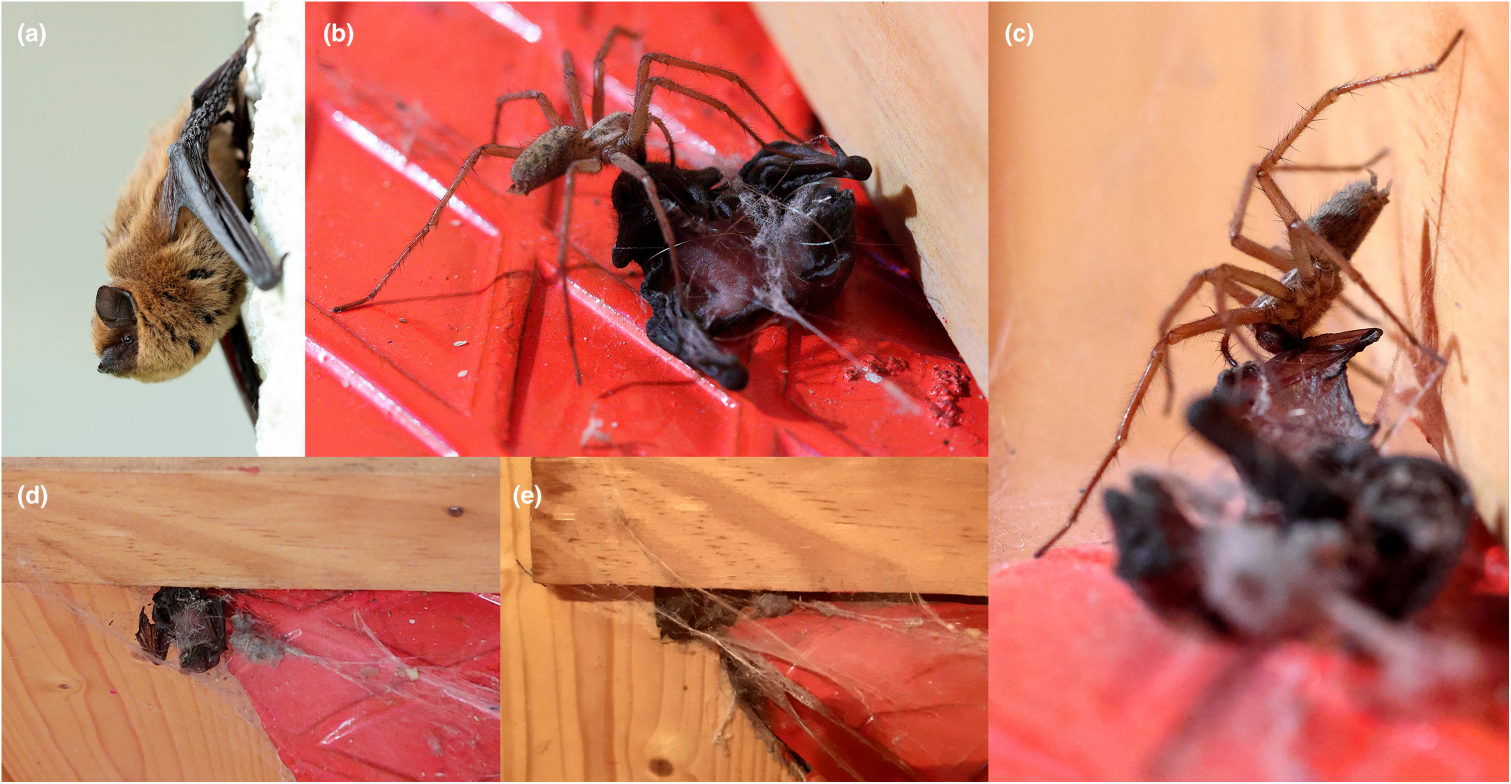
Photographs of the succession of events during the predatory event of a common pipistrelle bat (*Pipistrellus pipistrellus*) pup by a common house spider (*Eratigena* sp.) in NW Spain on 2 July 2023. (a) Adult common pipistrelle perched outside the maternity colony; (b) common house spider feeding on the tail membrane of the bat pup that is still alive; (c) house spider feeding on the left wing membrane of the already dead pup; (d) bat pup corpse at the entrance of the funnel web typical of this spider species; and (e) pup corpse dragged completely inside the funnel web.

The predation event in Spain occurred inside an inhabited building in the Western Cantabrian Mountains, NW Spain (42.723° N, 6.246° W). We report this event in detail, with photographs and video recording with a Canon R7 camera (Canon Inc, Japan) and a Samsung phone (Samsung Electronics, South Korea). At this site, a maternity colony of common pipistrelle (*Pipistrellus pipistrellus*; Figure 1a) uses the house during the summer months (ca. May to September), occupying the gap between the wooden roof rafters and the roofing slates. To confirm species identification, bats were acoustically recorded leaving the roost with an Echo Meter Touch 2 detector (Wildlife Acoustics Inc., United States) and identified following Russ (2021). The spider was identified as *Eratigena* sp. from videos and photographs and confirmed by specialists from the local Iberian Spider Group (Iberian Spiders, 2024; Nentwig et al., 2024). The event occurred on the evening of 2 July 2023. At 20:00 (UTC + 2 h), we detected the grounded bat pup on the floor inside the house, partially covered by spider web, and with a large domestic house spider (*Eratigena* sp., family Agelenidae) holding onto it (Video 1, Figure 1b). The bat pup was suspected to have fallen from the maternity roost through a gap above the wooden rafter structure of the ceiling. At the moment of the discovery, the pup was still alive and soon after, the spider was recorded feeding upon the tail membrane (Video 1, Figure 1b). At 21:19 the pup was immobile and was presumed dead, and the spider retreated into its web funnel under a staircase step ca. 20 cm away from the corpse. After a 20 min period, the spider emerged again and resumed feeding on the bat's left wing membrane (Video 1, Figure 1c) and did so continuously until 23:28. During the night we stopped monitoring the event, and by the next morning (07:22) the spider had dragged the dead bat pup to its funnel web (Figure 1d) and was consuming it. At 11:21 the bat was even deeper into the web and the spider continued eating it, and by 14:41 the bat corpse was completely inside the funnel mostly out of sight (Figure 1e) and it was not possible to continue monitoring the process.

**VIDEO 1:** Series of videos documenting the predatory event of a common pipistrelle bat (*Pipistrellus pipistrellus*) pup by a common house spider (*Eratigena* sp.) in NW Spain on 2 July 2023.

**FIGURE 2:**
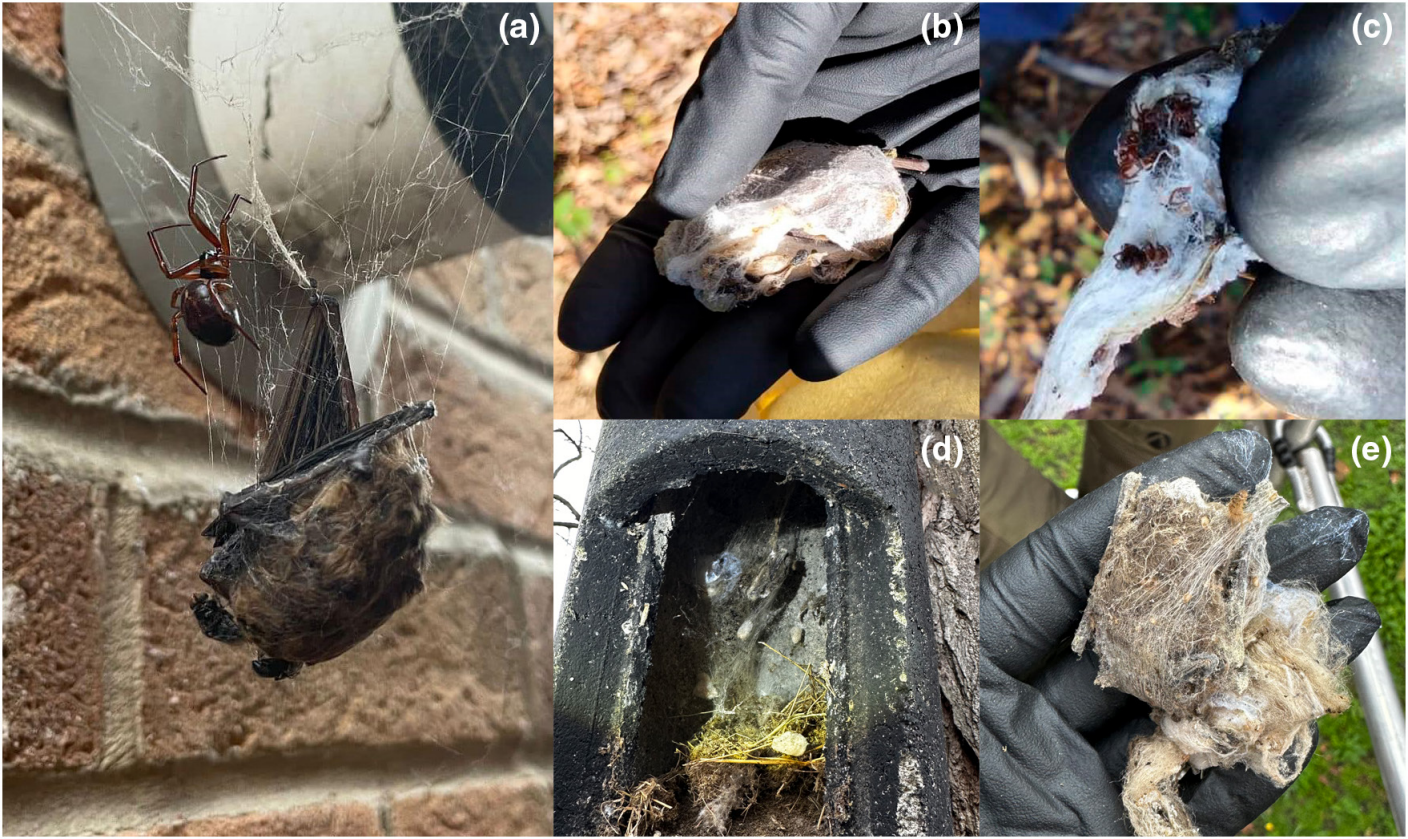
(a) Adult pipistrelle bat (*Pipistrellus* sp.) caught in the scaffold spider web of a noble false widow (*Steatoda nobilis*) on the exterior of a residential building in Taverham, UK, on 21 June 2023; (b) adult soprano pipistrelle (*Pipistrellus pygmaeus*) found completely wrapped and still alive in a wooden crevice bat box in Essex on 17 September 2022; (c) hatched spiderlings within the spider web that constrained the soprano pipistrelle; (d) adult pipistrelle bat (*Pipistrellus* sp.) found dead inside Schwegler 2FN bat box in London on 27 February 2024, wrapped completely in thick spider web; and (e) the same pipistrelle bat in hand, showing overall size and spider web wrapping.

The three accounts in the United Kingdom involve bats entangled in spider webs. On 21 June 2023, an adult pipistrelle bat (*P. pipistrellus*/*P. pygmaeus*) was recorded dead and entangled in a spider web, next to its potential predator, a female noble false widow spider (*Steatoda nobilis*, family Theridiidae; Figure 2a), on the side of a building in Taverham in Norfolk, UK (52.679° N, 1.211° E). Unfortunately, only rapid photographic documentation was possible, and the event was not tracked further. The spider was identified from photographs and confirmed by members of the British Arachnological Society (BAS, 2024; Nentwig et al., 2024; Vanuytven, 2021). On two other occasions, entangled bats were recorded inside a handmade wooden crevice bat box and a 2FN Schwegler bat box at two sites in the United Kingdom. These bat box checks were conducted as part of monitoring projects utilising boxes to survey bat species assemblages and different aspects of their ecology and behaviour (Rueegger, 2016). The first case was in a woodland in Little Baddow in Essex (51.732° N, 0.561° E) on 17 September 2022. A soprano pipistrelle (*P. pygmaeus*) was found wrapped in thick spider web inside the wooden bat box (Figure 2b). Upon removal of the spider web, the bat was observed to still be alive and apparently uninjured. Within the remains of the spider web, a significant number of bat droppings were found, and further inspection revealed the presence of tiny spiderlings (Figure 2c). The second case was in an urban area of Brompton in London (51.487° N, 0.193° W) on 27 February 2024. A dead adult pipistrelle bat (either *P. pipistrellus* or *P. pygmaeus*) was found tightly entangled in a thick spider web (Figure 2d,e). The bat was stiff and slightly discoloured, and it was not possible to determine how long it had been in there or dead for. On both occasions, the spiders were not present in the bat boxes and a species identification for the spider was not possible.

## 3 DISCUSSION

Our study reports four cases of spider predation on bats in Europe, ranging from direct predation to web entanglement resulting in bat mortality. The predation event recorded in Spain represents the first case of a spider capturing and consuming a bat known to the country, and the first documented event in Europe with clear evidence of immobilisation and consumption. Moreover, it is, to the best of our knowledge, the first record of bat predation by a spider of the family Agelenidae globally. One record is known of potential predation of a mammal by an Agelenid spider, where a mouse was killed in a spider funnel web, but no evidence of consumption was recorded (Nyffeler & Gibbons, 2022). Agelenid spiders are classified as occasional, rather than habitual vertebrate‐eaters (Nyffeler & Gibbons, 2022). Within the family, *Eratigena* house spiders are known for their large size and venomous capability, and the construction of funnel webs where they await prey (Vibert et al., 2016). Such structures lack sticky threads and require a fast response of the spider to successfully capture their prey hitting the web, which mainly consists of soft‐bodied arthropods such as butterflies, bees, wasps and flies (Nentwig, 1987). Newborn non‐volant bat pups are considerably vulnerable without their mothers, unable to thermoregulate (Racey & Entwistle, 2000) and with limited mobility to escape from predators (Dool & Uhl, 2022). This makes them easy to capture by ground‐dwelling predators when they accidentally fall from the roost. Consequently, the construction of funnel webs in the proximity of bat roosts could represent a valuable opportunity for spiders to prey on vulnerable newborn bats. Interestingly, the recording suggests the *Eratigena* spider preferentially targets the tail and wing membranes of the bat pup to feed on highly vascularised areas (Holbrook & Odland, 1978), perhaps offering easier access to nutrients (Toft, 2013).

In general, spiders of larger body size and strength are the ones that are able to subdue vertebrate prey. However, spiders of the Theridiid family, to which *Steodata nobilis* belongs, have been highlighted as an exception to this rule. Although their main food source are ants, bees and beetles (Nentwig, 1987), they are also able to capture and consume vertebrate prey despite their smaller size (Nyffeler & Gibbons, 2022). They compensate for their size by using potent venom that can paralyse and kill small vertebrates and construct extremely sticky and strong webs (Nyffeler & Gibbons, 2022). The false noble widow spider preys on European small mammals, including pygmy shrews (Dugon et al., 2023) and a disputed case of predation on bats (Dool & Uhl, 2022; Dunbar et al., 2022). We contribute a record of capture of an adult bat in the web of a false noble widow, but further research is needed to document these interactions and establish whether these spiders consume the bats they capture in their webs. We highlight here that our ability to confirm the spider species involved in these interactions relied on high‐resolution imagery, showcasing its potential to help document and understand elusive ecological dynamics (Carreira, 2024; Maritz & Maritz, 2020).

Our findings align with those most frequently described by Nyffeler and Knörnschild (2013), involving web‐building spiders, and aerial insectivorous vespertilionid species. Despite the limitations of not being able to conclusively monitor all the interactions, we report a clear predation event of the house spider killing the bat pup and consuming the remains. Therefore, European spiders can at least handle and process small vespertilionid bats as prey, and we cannot rule out that the spiders in bat boxes would prey on the bats they captured. The case of a soprano pipistrelle bat completely entangled in a thick web with hatching spiderlings, but still alive, implies that there had not been any attempt of consumption up to that point. Nonetheless, without intervention, the bat would have died, resulting in mortality through entanglement, as exemplified in the second bat box case. It is necessary to investigate these incidences further, to uncover the true nature and extent of these interactions and resolve whether the bats are actively detected, entangled, immobilised/killed (e.g., via venom injection) and/or consumed by the spiders. Our findings support that these interactions may be more prevalent than previously assumed (Nyffeler & Gibbons, 2022; Nyffeler & Knörnschild, 2013). In part, the darkness and inaccessibility of the areas regularly used both by bats and spiders (e.g., caves, rock and tree crevices, gaps in buildings) may explain the low number of observed predation events.

The complexity of spider predation and defence strategies enable complex predator–prey interactions with vertebrates, including flying mammals. Our study sheds light on the often‐overlooked interactions between bats and spiders in European ecosystems, particularly when the tables are turned and the presumed predator turns prey. By documenting these findings, we offer new insights into spider predation on bats and underscore the importance of continued research efforts into these largely undocumented ecological interactions, predator effects and trophic links.

## AUTHOR CONTRIBUTIONS

**Héctor Ruiz‐Villar:** Conceptualization (equal); investigation (lead); software (lead); visualization (lead); writing – original draft (equal); writing – review and editing (equal). **Cecilia Montauban:** Conceptualization (equal); writing – original draft (lead); writing – review and editing (equal). **Ana Pino‐Blanco:** Conceptualization (equal); writing – original draft (supporting); writing – review and editing (equal). **Elena Tena:** Conceptualization (lead); supervision (lead); writing – original draft (equal); writing – review and editing (equal).

## CONFLICT OF INTEREST STATEMENT

The authors declare that they have no competing interests.

## Data Availability

All data used for this manuscript are based on direct observations, and it is presented in the text and supplementary video, which are available to all readers, reviewers and editors.
